# SREBP1 site 1 protease inhibitor PF-429242 suppresses renal cell carcinoma cell growth

**DOI:** 10.1038/s41419-021-03999-9

**Published:** 2021-07-20

**Authors:** Tong-bing Wang, Mei Geng, Hua Jin, Ai-guo Tang, Hao Sun, Liu-zheng Zhou, Bin-hai Chen, Gang Shen, Qiang Sun

**Affiliations:** 1 Department of Urology, People’s Hospital of Yangzhong City, Yangzhong, China; 2 Department of Oncology, Rui Jin Hospital Affiliated to Shanghai Jiao Tong University School of Medicine, Shanghai, China; 3 The Child Health Care Department, Suzhou Ninth People’s Hospital, Suzhou, China; 4 Department of Urology, The affiliated Hospital of Jiangsu University, Zhenjiang, China; 5 Department of Urology, DUSHU Lake Hospital Affiliated to Soochow University, Suzhou, China; 6 Department of Nephrology, Affiliated Kunshan Hospital of Jiangsu University, Kunshan, China

**Keywords:** Targeted therapies, Renal cell carcinoma

## Abstract

Renal cell carcinoma (RCC) cells have increased lipogenesis and cholesterol synthesis. Sterol regulatory element-binding protein-1 (SREBP1) is cleaved by site 1 protease (S1P) to release the transcriptionally active amino-terminal domain. PF-429242 is a potent and competitive S1P inhibitor. We here tested its activity in RCC cells. In established and primary human RCC cells, PF-429242 potently inhibited cell proliferation, migration, and invasion. The S1P inhibitor provoked apoptosis activation in RCC cells. Furthermore, shRNA-mediated S1P silencing or CRISPR/Cas9-induced S1P knockout led to RCC cell growth inhibition and apoptosis activation. Conversely, ectopic overexpression of SREBP1 or S1P augmented RCC cell proliferation and migration. Daily *i.v*. injection of a single dose of PF-429242 robustly inhibited RCC xenograft growth in severe combined immunodeficiency mice. Additionally, intratumoral injection of S1P shRNA lentivirus inhibited RCC xenograft growth in mice. SREBP1, S1P, and its target gene low density lipoprotein receptor (LDLR) were significantly elevated in human RCC tissues. These results suggest that targeting S1P by PF-429242 inhibited RCC cell growth in vitro and in vivo.

## Introduction

Renal cell carcinoma (RCC) is the most common renal malignancy and it accounts for over 2% of all human cancers [1, 2]. Significant progresses have been achieved in current RCC treatments, including radical surgical resection and molecularly-targeted therapies [3–5]. However, 25% of RCC patients can still develop disease progression or metastasis [6, 7]. This could be due to the molecular heterogeneity of RCC [4]. Therefore, it is extremely important to identify novel prognostic biomarkers and therapeutic targets for this devastating disease. Thus, individualized treatments that assess therapeutic responses and optimize patients prognosis could be developed [4, 5, 8–10].

The Cancer Genome Atlas (TCGA) has revealed that RCC patients with poor prognosis have characteristic metabolic remodeling that is similar to Warburg metabolic phenotypes, including glycolysis, glutamine dependent lipogenesis, and decreased AMPK (AMP-activated protein kinase) activation [3]. Metabolic remodeling plays a vital role in RCC tumorigenesis and progression [3, 11, 12]. However, the underlying signaling mechanisms are still largely unknown.

Sterol regulatory element-binding protein (SREBP) family proteins, SREBP1, and SREBP2 are basic helix-loop-helix-leucine zipper transcription factors [13–16]. SREBP1 and the lipogenic genes are elevated in RCCs, which are associated with advanced tumor stages and poor prognosis [3]. Lee and colleagues have demonstrated that SREBP1 is upregulated in RCC [11]. Conversely, SREBP1 inhibition by genetic and pharmacological strategies inhibited RCC cell growth [11]. Therefore, targeting SREBP1 could be a novel therapeutic strategy against RCC.

SREBPs are cleaved at the N-terminal domain and are translocated to cell nuclei. Thereafter, activated SREBPs bind directly to the sterol regulatory element DNA sequence (TCACNCCAC) to promote the expression and synthesis of enzymes for lipogenesis and cholesterol synthesis [13–16]. Two independent and site-specific proteolytic cleavages are required to release the transcriptionally active amino-terminal domain of SREBPs. The two are carried out by two different proteases, including site 1 protease (S1P) and site 2 protease (S2P) [12–14, 17]. PF-429242 is a reversible and competitive S1P inhibitor with a relatively low IC50 (175 nM) [18, 19]. The current study analyzed the potential effect of PF-429242 in human RCC cells.

## Materials and methods

### Reagents and chemicals

PF-429242 was provided by Adooq (Nanjing, China). Cell culture reagents were obtained from Sigma-Aldrich (St. Louis, Mo). Anybodies for acetyl-CoA synthetase (ACS), pituitary tumor transforming gene 1 (PTTG1)/Securin (#13445), cleaved-caspase-3 (#9664), cleaved-caspase-9 (#20750), cleaved-poly (ADP-ribose) polymerase (PARP) (#5625), and β-tubulin (#15115) were purchased from Cell Signaling Tech (Beverly, MA). Anti-LDL receptor (LDLR) antibody (ab30532), anti-SREBP1 antibody (ab191857), and anti-S1P antibody (ab59870) were obtained from Abcam (Shanghai, China). Viral constructs, primers, and other sequences were provided by Genechem (Shanghai, China). TRIzol reagents and other RNA assay agents as well as transfection reagents were provided by Thermo-Fisher Invitrogen Co. (Shanghai, China).

### Cell culture

The clear cell RCC (ccRCC) cell line A498, the HK-2 immortalized tubule epithelial cells [20, 21], and the primary human renal epithelial cells (“renal Epi”) were from Dr. Zhu at Soochow University [22]. Primary human RCC cells, derived from three patients (namely “RCC1/2/3”) were also provided by Dr. Zhu [22]. Patient RCC1: Male, 69, ccRCC, CK7 (−), CD10 ( + ), CK20 (−), CD34 ( + ), EMA ( + ), Ki67 (+), Vimentin (+); Patient RCC2: Female, 82, ccRCC CK7 (−), CD10 ( + ), CK20 (−), CD34 ( + ), EMA ( + ), Ki67 (+), Vimentin (+); Patient RCC3: Male, 67, ccRCC, CK7 (-), CD10 ( + ), CK20 ( + ), EMA ( + ), Ki67 (+), Vimentin (+). The detailed protocols of culturing RCC cells and epithelial cells were described previously [23, 24]. Each enrolled participant provided written informed consent. Cells were subjected to mycoplasma and microbial contamination examination on a regular basis. Every three months, STR profiling, population doubling time, morphology were examined to verify the origin of cells.

### Human tissues

A total of eight different patients with primary RCC were enrolled, who underwent RCC nephrectomy. Patient characteristics are summarized in Table 1. RCC “tumor tissues (“T”) and cancer-surrounding normal tissues (“N”)” were acquired and separately carefully. Fresh tissues were washed, minced, and homogenized, and tissue lysates were stored in liquid nitrogen. The quantitative reverse transcriptase PCR (“RT-qPCR”) and Western blotting assays were performed to test SREBP1-S1P pathway genes in the lysates. The written informed consent was obtained from each patient, who received no prior chemotherapy or targeted treatments before surgeries. The protocols were approved by the Ethics Committee of Affiliated Kunshan Hospital of Jiangsu University (BR2016018), according to the Declaration of Helsinki.

**Table 1:** Patient characteristics.

Patient No.	Type	R/L	Gender	Age	Ki-67%	Stage
Patient-1	ccRCC	R	F	62	30%	IV
Patient-2	ccRCC	R	M	41	20%	III
Patient-3	ccRCC	R	M	49	1%	II
Patient-4	ccRCC	L	M	70	1%	III
Patient-5	ccRCC	R	M	56	5%	II
Patient-6	ccRCC	R	M	67	10%	III
Patient-7	ccRCC	R	F	82	10%	II
Patient-8	ccRCC	R	M	69	3%	III

*ccRCC* clear cell renal cell carcinoma, *R* Right, *L* Left, *F* female, *M* Male.

### CCK-8 assay

Cells were seeded into 96-well plates (at 4, 000 cells per well). Following the applied treatment, 10 μL CCK-8 reagent was added to each well and cells were incubated for 3 h in a 5% CO_2_ incubator at 37 °C. CCK-8’s optical density (OD at 450 nm) was tested under a microplate reader.

### Colony formation assay

RCC cells were seeded into 10-cm tissue culture dishes (at 3 × 10^4^ cells per dish). Cells were cultured for a total of 10 days. PF-429242-containing medium was renewed every two days. Cells were then washed with PBS and fixed with 100% methanol. Afterward, 0.5% crystal violet was utilized to stain the viable colonies. Viable RCC cell colonies were counted manually.

### Cell cycle studies

Cells were seeded into six-well plates (1 × 10^5^cells per well). Following the applied treatment, cells were fixed with 70% ethanol at − 20 °C overnight and were then incubated with RNase for 30 min at 37 °C. Subsequently, cells were stained with propidium iodide (PI, 5 μg/mL, Invitrogen Thermo-Fisher) at room temperature under the dark for 30 min. Cells were then subjected to fluorescence-activated cell sorting (FACS) at 488 nm excitation wavelength.

### Lactate dehydrogenase (LDH) assay

Cells were seeded into 12-well plates (at 5 × 10^4^ cells per well). Following the applied treatment, the LDH activity was measured in both cell lysates and supernatants using in vitro LDH assay kit (Sigma) in accordance with the manufacturer’s protocols. The LDH absorbance was determined at 490 nm. The % LDH release from the cells was calculated by dividing absorbance of the culture supernatants to absorbance of supernatant plus cells lysate.

### “Transwell” assays

Using the previously-described protocol [25, 26], RCC cells (3 × 10^4^ cells per condition), cultured in 250 μL of serum-free medium, were seeded onto the upper surface of “Transwell” (12 μm pore) chambers. Complete medium with 12% FBS was added to the lower compartments. Cells were allowed to migrate for 24 h. Afterward, migrated cells on the lower surface were fixed, stained, and counted [27]. When examining cell invasion in vitro, “Matrigel” (Sigma) was added on the surface.

### Caspase activity assay

As described [22], cells were initially seeded into six-well plates (at 1.2 × 10^5^cells per well). Following the applied treatment, 20 μg of cytosolic extracts were utilized to examine caspase-3/−9 activity using a previously described protocol [28]. The caspase-3/−9 substrates were conjugated with7-amido-4-(trifluoromethyl)-coumarin (AFC) [28]. AFC intensity was examined in a Fluoroskan machine [28].

### Annexin V FACS and other apoptosis assay

As reported [28], cells with the applied treatments were washed and stained with Annexin V-FITC (5 μg/mL, Invitrogen Thermo-Fisher) and PI (5 μg/mL). Cells were then examined under a Becton-Dickinson FACS machine. Annexin V-positive cells were sorted as apoptotic cells. The detailed protocols of other apoptosis-related assays, including nuclear TUNEL staining, single-strand DNA (ssDNA) ELISA (testing DNA breaks), and JC-1 dye assay of mitochondrial depolarization, was described in detail in elsewhere [22, 29].

### EdU staining

As reported [29, 30], cells were seeded into six-well plates (1 × 10^5^cells per well). Follow applied treatments, cells were stained with EdU (Invitrogen, 5 μM). Afterward cell nuclei were co-stained with DAPI and visualized under a fluorescent microscope (Leica). In each condition, at least 1000 cells from five random views (1 × 100 magnification) were counted to calculate the average EdU ratio (EdU/DAPI × 100%).

### Western blotting

The detailed protocols for Western blotting were described elsewhere [22, 31]. The same set of lysates were run in parallel (“sister”) gels to test different proteins when necessary. Data quantification was carried out using ImageJ software (NIH).

### RT-qPCR

In brief, TRIzol reagents were applied to extract total RNA, which were reversely transcripted to cDNA. An SYBR Green PCR kit (Applied Biosystems) was utilized for RT-qPCR assays under the ABI 7900HT Real-Time PCR system [32]. *GAPDH* was tested as the internal control. Quantifications of targeted mRNAs were via a 2^−∆∆*C*t^ method. The mRNA primers were listed in Table 2.

**Table 2:** Sequences utilized in this study.

qPCR primers		
Genes	Forward (5’−3’)	Reverse (5’−3’)
***LDLR***	GAATCTACTGGTCTGACCTGTCC	GGTCCAGTAGATGTTGCTGTGG
***PTTG1***	GCTTTGGGAACTGTCAACAGAGC	CTGGATAGGCATCATCTGAGGC
***ACS***	GGTGACCAAGTTCTACACAGCAC	GTTCACCCACTGTGCCTAACAC
***S1P***	CTACTATGGAGGAATGCCGACAG	CTCCGTTCTGTGGCAAATAGGG
***GAPDH***	GTCTCCTCTGACTTCAACAGCG	ACCACCCTGTTGCTGTAGCCAA
**sgRNA sequences (5’−3’)**		
**S1P sgRNA-1**	GGCGTGACCCGTTTGATGTT (Target DNA Sequence)	

### Total cholesterol assay

Cells were seeded into 96-well plates (at 4000 cells per well). Following the applied treatments, total cholesterol levels were quantified using a cholesterol oxidase probe by a cholesterol assay kit (Abcam, Beijing, China). The absorbance of each well was detected at 570 nm.

### Ectopic overexpression of S1P or SREBP1

The full-length *S1P* cDNA and *SREBP1* cDNA were provided by Genechem and individually annealed into a GV280 vector (hU6-MCS-Ubiquitin-EGFP-IRES-puromycin, Genechem). The construct was then transfected to HEK-293T cells along with lentivirus package plasmids (Genechem) to generate S1P-expressing lentivirus (LV-S1P) and SREBP1-expressing lentivirus (LV-SREBP1). Thereafter, viruses were enriched (to 2 × 10^8^transducing units/mL), filtered and added to cultured RCC cells. Cells were cultured in a polybrene-containing complete medium. Stable cells were established via adding puromycin. S1P or SREBP1overexpression in stable cells was verified by RT-qPCR and Western blotting assays. Control cells were transduced with an empty vector (GV280).

### S1P and SREBP1 shRNA

RCC cells were seeded into six-well tissue culture plates at 50-60% confluence and were cultured in a polybrene-containing complete medium. SREBP1 shRNA lentiviral particles (sc-44327-V, Santa Cruz Biotech, Santa Cruz, CA) or S1P shRNA lentiviral particles (sc-36496-V, Santa Cruz Biotech) were added to RCC cells for 48 h. Puromycin was added to the medium. After five passages, stable cells were established. Knocking-down of targeted protein was verified by RT-qPCR and Western blotting assays. Control cells were transduced with scrambled control shRNA lentiviral particles (“shC”, Santa Cruz Biotech).

### S1P knockout

A CRISPR/Cas9-S1P-KO-GFP construct was provided by Genechem. The targeted DNA sequence was listed in Table 2. RCC cells were seeded into six-well plates (at 50–60% confluence) and culture in complete medium. Cells were transfected with the CRISPR/Cas9 S1P KO construct via Lipofectamine 3000 protocol. The transfected cells (GFP positive) were further sorted by FACS and distributed into 96-well plates. Single stable cells were established and screened for *S1P* KO by RT-qPCR.

### Xenograft assay

Severe combined immunodeficiency (SCID) mice (5–6 week old, 18–18.5 g weight, half male half female) were provided by the Experimental Animal Center of Soochow University (Suzhou, China). Human RCC cells, in Matrigel-containing serum-free medium, were *s.c*. injected to the flanks of SCID mice (at eight million cells per mouse). RCC tumor xenografts were established with 20 days (volume around 100 mm^3^). The tumor-bearing mice were randomly assigned into different groups and were subjected to applied treatments. Mice body weights and tumor volumes were tested as described [28]. The animal studies were approved by the Institutional Animal Care and Use Committee (IACUC) and Ethics Committee of Affiliated Kunshan Hospital of Jiangsu University.

### Statistics

Quantitative data with normal distribution were expressed as mean ± standard deviation(SD). Statistical comparisons between multiple groups were performed via one-way ANOVA (SPSS 23.0, SPSS Co., Chicago, CA). When comparing two treatment groups, the two-tailed unpaired *T*-test (Excel 2007) was carried out. *P* values < 0.05 were statistically significant.

## Results

### PF-429242 inhibits RCC cell growth

**Fig. 1:**
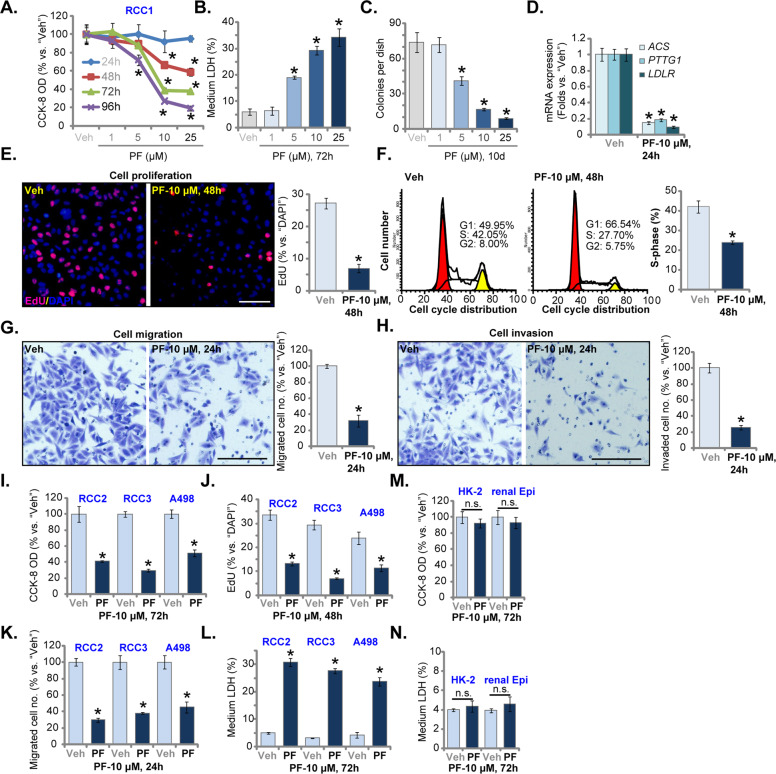
PF-429242 inhibits RCC cell growth. Primary human RCC cells (“RCC1/RCC2/RCC3”, **A**–**L**), A498 cells (**I**–**L**), HK-2 tubular epithelial cells (**M**, **N**) or the primary renal epithelial cells (“renal Epi”) (**M**, **N**) were treated with the applied concentration of PF-429242 (“PF”, same for all Figures) and further cultured for applied time periods; Cell number, cell death, colony formation, and proliferation, cell cycle progression as well as cell migration and invasion were tested by the appropriate assays mentioned in the text. Expression of listed mRNAs was tested by RT-qPCR assays (**D**). For each assay, *n* = 5. Data were expressed as the mean ± standard deviation (SD). “Veh” stands for 0.1% of DMSO (same for all Figures). *P < 0.05 vs. “Veh” group (**A**–**L**). “n.s.” stands for no statistical difference (**M**, **N**). In this figure, experiments were repeated three times, and similar results were obtained each time. Scale bar = 100 μm (**E**, **G**, and **H**).

The primary human RCC cells, RCC1 (from Dr. Zhu [22]), were cultured in FBS-containing complete medium and treated with PF-429242 (at gradually-increased concentrations: 1–25 μM). Cells were cultured for 24–96 h. CCK-8 assays demonstrated that PF-429242 treatment led to less viable RCC1 cells (Fig. 1A). PF-429242 displayed a concentration-dependent manner, being significant at 5–25 μM. It was however ineffective at 1 μM (Fig. 1A). Furthermore, PF-429242 required at least 48 h to significantly reduce viable RCC1 cell number (Fig. 1A), showing a time-dependent response (Fig. 1A). Medium LDH release assay results confirmed that PF-429242, at 5–25 μM, induced significant RCC1 cell death (Fig. 1B). Colony formation assay results in Fig. 1C demonstrated that PF-429242 (5–25 μM) decreased the number of viable RCC1 cell colonies. At 1 μM PF-429242 failed to significantly induce medium LDH release (Fig. 1B) and inhibit colony formation (Fig. 1C). Since 10 μM of PF-429242 induced significant activity in RCC cells, this concentration was chosen for the following studies.

In RCC1 cells, treatment with PF-429242 (10 μM, 24 h) downregulated SREBP1-S1P dependent genes, including acetyl-CoA synthetase (ACS), PTTG1 and low-density lipoprotein receptor (LDLR) [13, 14, 17, 33] (Fig. 1D). These results implied that PF-429242 blocked SREBP1-S1Pcascade. Results in Fig. 1E showed that PF-429242 (10 μM) decreased nuclear EdU incorporation in RCC1 cells. Cell cycle studies showed that G1 phase percentage cells were significantly increased with PF-429242 treatment, but S- and G2-phase cells were decreased (Fig. 1F). Thus, the S1P inhibitor resulted in proliferation inhibition and G1-S arrest in RCC1 cells. “Transwell” assay results in Fig. 1G showed that PF-429242 (10 μM, 24 h) decreased the number of migrated RCC1 cells. Cell invasion, tested by “Matrigel Transwell” assay, was significantly inhibited as well (Fig. 1H). For the “Transwell” and “Matrigel Transwell” assays, RCC1 cells were incubated with PF-429242 for 24 h (Fig. 1G and H), and no significant cytotoxicity was detected (Fig. 1A).

In RCC2 and RCC3 cells, the primary RCC cells derived from two other RCC patients (from Dr. Zhu [22]), as well as in the established A498 cells, PF-429242 (10 μM) treatment similarly decreased the number of viable cells (Fig. 1I). It also inhibited cell proliferation (by recording EdU-positive nuclei ratio, Fig. 1J) and migrated cell number (“Transwell” assays, Fig. 1K). The S1P inhibitor-induced medium LDH release in the RCC cells (Fig. 1L). Conversely, in the immortalized HK-2 tubular epithelial cells [34, 35] and primary renal epithelial cells (“renal Epi”), PF-429242 treatment (10 μM, 72 h) failed to induce CCK-8 OD reduction (Fig. 1M) and cell death (medium LDH release, Fig. 1N).

### PF-429242 provokes apoptosis activation in RCC cells

**Fig. 2:**
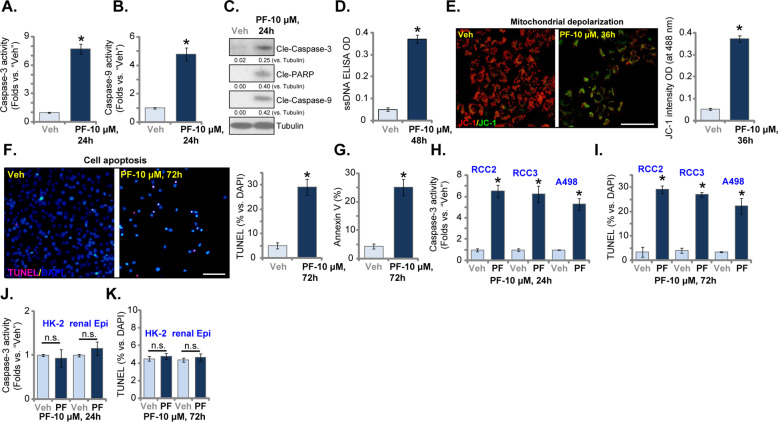
PF-429242 provokes apoptosis activation in RCC cells. Primary RCC cells (“RCC1/RCC2/RCC3”, **A**–**I**), A498 cells (**H**, **I**), HK-2 tubular epithelial cells (**J**, **K**) or the primary renal epithelial cells (“renal Epi”) (**J**, **K**) were treated with the applied concentration of PF-429242 and cultured for applied time periods, caspase activation (**A**–**C**, **H**, and **J**) and apoptosis induction (**F**, **G**, **I**, and **K**), DNA breaks (ssDNA ELISA OD, **D**), and mitochondrial depolarization (JC-1 assay, **E**) were tested by the appropriate assays mentioned in the text, and results were quantified. For each assay, *n* = 5. Data were expressed as the mean ± standard deviation (SD). * *P* < 0.05 vs. “Veh” group (**A**–**I**). “n.s.” stands for no statistical difference (**J**, **K**). In this figure, experiments were repeated three times, and similar results were obtained each time. Scale bar = 100 μm (**E**, **F**).

Proliferation inhibition and cell cycle arrest could induce RCC cell apoptosis [22–24], we therefore analyzed the potential effect of PF-429242 on cell apoptosis. As shown, the caspase-3 activity (Fig. 2A) and the caspase-9 activity (Fig. 2B) were both significantly increased in PF-429242 (10 μM, 24 h)-treated RCC1 cells. The S1P inhibitor-induced cleavages of caspase-3, caspase-9, and PARP (Fig. 2C). Additionally, increased single-strand DNA (ssDNA) accumulation was detected in RCC1 cells after PF-429242 treatment (Fig. 2D). Furthermore, PF-429242 induced mitochondrial depolarization, causing JC-1 green monomers accumulation in RCC1 cells (Fig. 2E). These results implied the activation of the caspase-3-dependent mitochondrial apoptosis pathway by PF-429242 in RCC1 cells [36–38].

Importantly, PF-429242 induced apoptosis activation in RCC1 cells, as the TUNEL-positive nuclei ratio (Fig. 2F) and Annexin V-positive staining ratio (Fig. 2G) were significantly increased after PF-429242 treatment. In A498, RCC2, and RCC3 cells, PF-429242 (10 μM) similarly increased caspase-3 activity (Fig. 2H) and the TUNEL-positive nuclei ratio (Fig. 2I). However, in HK-2 tubular epithelial cells and primary renal epithelial cells, PF-429242 (10 μM) treatment failed to induce significant caspase-3 activation (Fig. 2J) and apoptosis activation (Fig. 2K). Thus PF-429242 provoked apoptosis activation in RCC cells.

### S1P silencing or knockout inhibits RCC cell growth

**Fig. 3:**
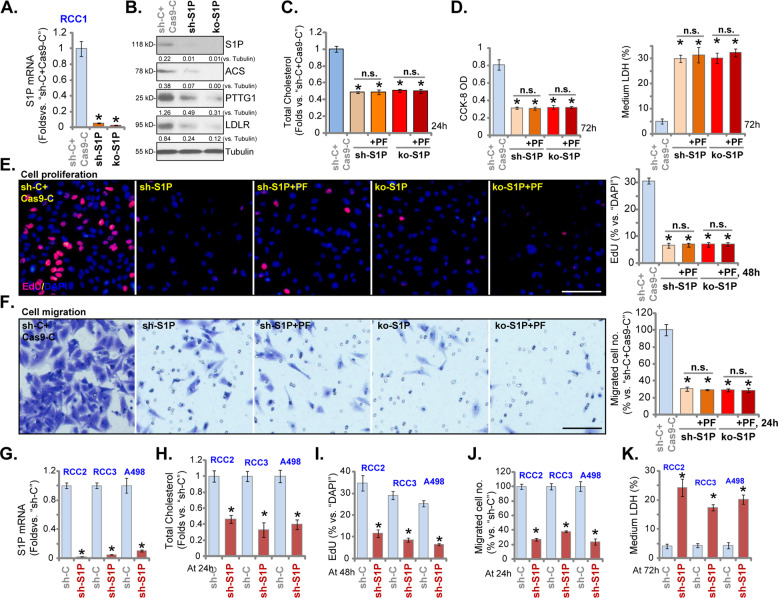
S1P silencing or knockout inhibits RCC cell growth. Stable RCC1 cells expressing S1P lentiviral shRNA (“sh-S1P” cells) or the CRSPR/Cas9-S1P-KO-GFP construct (“ko-S1P” cells) were established, control cells were transduced with scrambled control shRNA plus CRSPR/Cas9 empty vector (“sh-C + Cas9-C”); Expression of listed genes was shown (**A** and **B**); Cells were then treated with or without PF-429242 (10 μM), and cultured for applied time periods, total cholesterol levels were tested (**C**); Viable cell number, cell death, proliferation, and migration were tested by CCK-8 (**D**), LDH release (**D**), nuclear EdU staining (**E**) and “Transwell” (**F**) assays, respectively, and results were quantified.RCC2, RCC3, and A498 cells, expressing S1P lentiviral shRNA (“sh-S1P” cells) or scramble control shRNA (“sh-C”), were established and *S1P* mRNA expression was shown (**G**); Cells were further cultured for applied time periods, total cholesterol levels were tested (**H**); Cell proliferation, migration, and cell death were tested by nuclear EdU staining (**I**), “Transwell” (**J**) and medium LDH release (**K**) assays, respectively. For each assay, *n* = 5. Data were expressed as the mean ± standard deviation (SD).* *P* < 0.05 vs. “sh-C + Cas9-C”/“sh-C” group. “n.s.” stands for no statistical difference (**C**–**F**). In this figure, experiments were repeated three times, and similar results were obtained each time. Scale bar = 100 μm (**E** and **F**).

PF-429242 is an S1P inhibitor. We therefore hypothesized that S1P depletion should mimic PF-429242-induced activity in RCC cells. S1P shRNA lentiviral particles were transfected to RCC1 cells. Stable sh-S1P cells were established by puromycin selection. Furthermore, a CRSPR/Cas9-S1P-KO-GFP construct was transduced to RCC1 cells. Stable cells were established following FACS sorting. These cells were named as the ko-S1P cells. RT-qPCR assay results in Fig. 3A demonstrated that *S1P* mRNA decreased over 95% in sh-S1P cells and ko-S1P cells. S1P protein depletion was detected as well (Fig. 3B). Protein expression of SREBP1-S1P-dependent genes, ACS, PTTG1, and LDLR (Fig. 3B), as well as total cholesterol levels (Fig. 3C) were downregulated in RCC1 cells with S1P shRNA or S1P KO. S1P silencing or KO decreased viable cell number (Fig. 3D) and induced cell death (medium LDH release, Fig. 3D). RCC1 cell proliferation (by recording nuclei EdU ratio, Fig. 3E) and migration (Fig. 3F) were largely inhibited by S1P silencing or KO.

Importantly, in sh-S1P cells and ko-S1P RCC1 cells, adding PF-429242 (10 μM) failed to further alter total cholesterol levels (Fig. 3C) and cell death (Fig. 3D), as well as cell proliferation (Fig. 3E) and migration (Fig. 3F). Thus, S1P inhibition should be the primary mechanism of PF-429242-induced actions in RCC cells.

In RCC2, RCC3 and A498 cells, transfection of S1P shRNA lentiviral particles resulted in robust *S1P* mRNA downregulation (Fig. 3G), while reducing total cholesterol levels (Fig. 3H). S1P shRNA inhibited cell proliferation (decreased EdU-positive nuclei ratio, Fig. 3I) and the number of migrated cells (“Transwell” assays, Fig. 3J), and provoking cell death (LDH assay, Fig. 3K) in RCC cells.

### S1P overexpression promotes RCC cell proliferation and migration

**Fig. 4:**
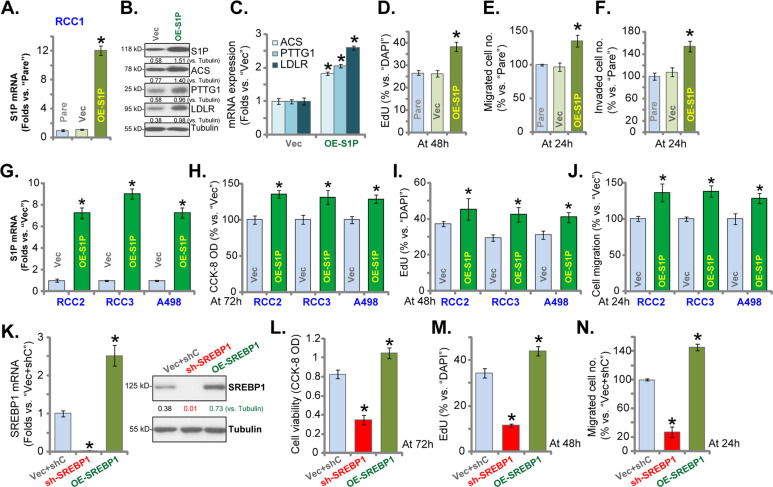
S1P overexpression promotes RCC cell proliferation and migration. Primary RCC cells (“RCC1/RCC2/RCC3”) or A498 cells, bearing the lentiviral S1P expression construct (“OE-S1P”) or empty vector (“Vec”), were established, expression of listed genes in these cells and parental control cells (“Pare”) was shown (**A**–**C** and **G**). Cells were further cultured for applied time periods, CCK-8 OD (**H**), proliferation (by recording nuclear EdU ratio, **D** and **I**), migration, and invasion (“Transwell” assay, **E**, **F**, and **J**) were tested. RCC1 cells bearing SREBP1 shRNA (“sh-SREBP1”) or SREBP1-expressing construct (OE-SREBP1) were established, control cells were transduced with scrambled control shRNA plus empty vector (“Vec+shC”), expression of *SREBP1* mRNA and protein was shown (**K**). Cells were further cultured for applied time periods, viable cell number (**L**), proliferation (**M**), and migration (**N**) were tested similarly. For each assay, *n* = 5. Data were expressed as the mean ± standard deviation (SD). *P < 0.05 vs. “Vec”/“Vec+shC” group. In this figure, experiments were repeated three times, and similar results were obtained each time.

We further hypothesized that ectopic S1P overexpression might be able to enhance RCC cell proliferation and migration. A lentiviral S1P-expression construct, LV-S1P, was transduced to RCC1 cells. Stable cells were established via puromycin selection (OE-S1P cells). RT-qPCR assay results in Fig. 4A demonstrated that *S1P* mRNA increased over ten folds in OE-S1P cells. S1P protein was increased as well (Fig. 4B). mRNA (Fig. 4C) and protein (Fig. 4B) levels of SREBP1-dependent genes, including *ACS*, *LDLR,* and *PTTG1*, were increased in the OE-S1P cells. Ectopic overexpression of S1P augmented RCC1 cell proliferation by increasing the EdU-positive nuclei ratio (Fig. 4D). “Transwell” assay results demonstrated that S1P overexpression enhanced RCC1 cell migration and invasion (results were quantified in Fig. 4E and F).

LV-S1P was also transduced to RCC2, RCC3, and A498 cells, and stable cells were established via selection by puromycin (“OE-S1P” cells), with*S1P* mRNA expression robustly increased (Fig. 4G). Ectopic overexpression of S1P increased the number of viable cells (Fig. 4H), cell proliferation (increased nuclear EdU ratio, Fig. 4I), and migrated cells (Fig. 4J).

SREBP1 should exert similar actions in RCC cells as S1Pdid. Therefore, RCC1 cells were transduced with either lentiviralSREBP1 shRNA or SREBP1-expressing construct. Stable cells were established: sh-SREBP1 cells and OE-SREBP1 cells. *SREBP1* mRNA and protein expression was silenced in sh-SREBP1 cells, but was elevated in OE-SREBP1 cells (Fig. 4K). Functional studies demonstrated that RCC1 cell number (Fig. 4L), cell proliferation (nuclear EdU ratio, Fig. 4M), and migration (Fig. 4N) were inhibited by SREBP1 shRNA, but augmented with SREBP1overexpression. These results further confirmed that the SREBP1-S1P axis is important for RCC cell growth.

### PF-429242 inhibits subcutaneous RCC xenograft growth in mice

**Fig. 5:**
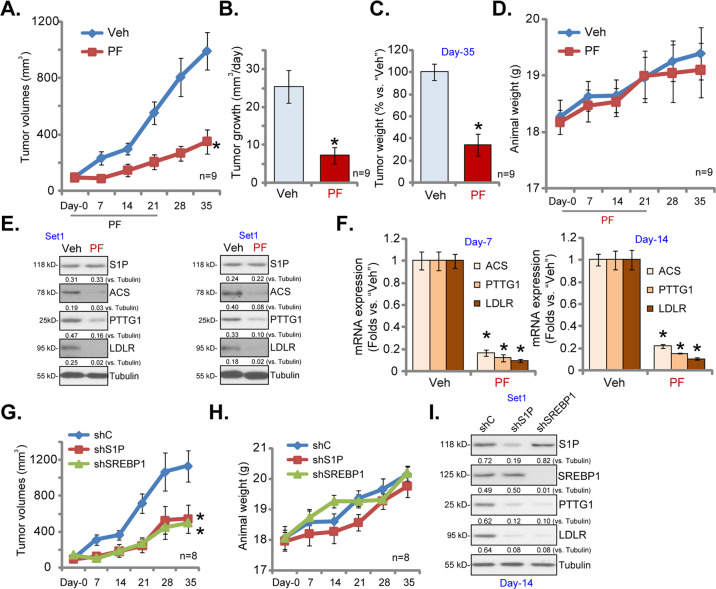
PF-429242 inhibits subcutaneous RCC xenograft growth in mice. RCC1 xenograft-bearing SCID mice were subjected to *i.v*. injection of PF-429242 (“PF”, at 10 mg/kg body weight, daily, for 21days) or vehicle control (“Veh”), tumor volumes (**A**) and mice body weights (**D**) were recorded every seven days; The estimated daily tumor growth, in mm^3^ per day, was calculated using the described formula (**B**). On Day-35 tumors of the two groups were separated through surgery and individually weighted (**C**). On Day-7 and Day-14, one tumor of each group was isolated and homogenized in tissue lysis buffer, expression of listed genes was shown (**E** and **F**). RCC1 xenograft-bearing SCID mice were subjected to intratumoral injection of SREBP1 shRNA lentivirus (“shSREBP1”), S1P shRNA lentivirus (“shS1P”) or scramble control shRNA lentivirus (“shC”), daily for five days, tumor volumes (**G**) and mice body weights (**H**) were recorded every seven days; At Day-14, one tumor of each group was isolated and homogenized in tissue lysis buffer, expression of listed proteins was shown (**I**). Data were expressed as the mean ± standard deviation (SD).*P < 0.05 vs. “Veh”/ “shC” group.

To study the activity of PF-429242 in vivo, we employed an RCC xenograft mice model. RCC1 primary cells were *s.c*. injected to the flanks of SCID mice. Within 20 days, RCC1 xenografts were established with the tumor volume close to 100 mm^3^ (labeled “Day-0”). RCC1 xenografts-bearing SCID mice were then randomly assigned into two groups (nine mice per group, *n* = 9). One group received PF-429242 *i.v*. injection (at 10 mg/kg body weight, daily, for 21days, dissolved in PBS). The other group was administrated with vehicle control. The weekly tumor growth curve results in Fig. 5A demonstrated that PF-429242 *i.v*. injection potently inhibited RCC1 xenograft growth in SCID mice. The volumes of RCC1 xenografts with PF-429242 administration were significantly lower than those with vehicle treatment (Fig. 5A). The estimated daily tumor growth was calculated using the established formula: (Tumor volume at Day-35—Tumor volume at Day-0)/35 [22, 23, 29]. RCC1 xenograft growth was potently inhibited following PF-429242 injection (Fig. 5B). At the end date of the experiment (Day-35), tumors were carefully separated through surgery and were weighted individually. RCC1 xenografts with PF-429242 administration were significantly lighter than the vehicle-treated tumors (Fig. 5C). Mice body weights were not significantly different between the two groups (Fig. 5D), indicating that mice were well-tolerated to the PF-429242 treatment.

At Day-7 and Day-14, one xenograft tumor of each group was isolated and homogenized (using tissue lysis buffer). Western blotting assay results in Fig. 5E confirmed dramatic downregulation of ACS, PTTG1, and LDLR proteins in PF-429242-treated tumor lysates. S1P protein expression was however unchanged (Fig. 5E). S1P-dependent genes, including *ACS*, *PTTG1,* and *LDLR*, were decreased as well in PF-429242-treated tumor tissues (Fig. 5F). Therefore, PF-429242 administration downregulated ACS, PTTG1, and LDLR in RCC1 xenografts.

Next RCC1 xenografts-bearing SCID mice were subjected to intratumoral injection of SREBP1 shRNA lentivirus (“shSREBP1”) or S1P shRNA lentivirus (“shS1P”). Viruses injection was performed daily for five consecutive days (Day-0 to Day-4). Control mice were treated with scramble control shRNA lentivirus (“shC”). Tumor growth curve results in Fig. 5G demonstrated that the growth of RCC1 xenografts was significantly inhibited after injection of SREBP1 shRNA viruses or S1P shRNA viruses. The mice body weights were not significantly different between the three groups (Fig. 5H).

At Day-14, one tumor of each group was isolated, and a total of three tumors were analyzed. The applied shRNA led to significant downregulation of target proteins (S1P and SREBP1) in tumor lysates (Fig. 5I). ACS and PTTG1 were downregulated in SREBP1-silenced and S1P-silenced tumor lysates (Fig. 5I). Therefore, SREBP1 or S1P silencing inhibited RCC xenograft growth in mice.

### SREBP1-S1P upregulation in human RCC

**Fig. 6:**
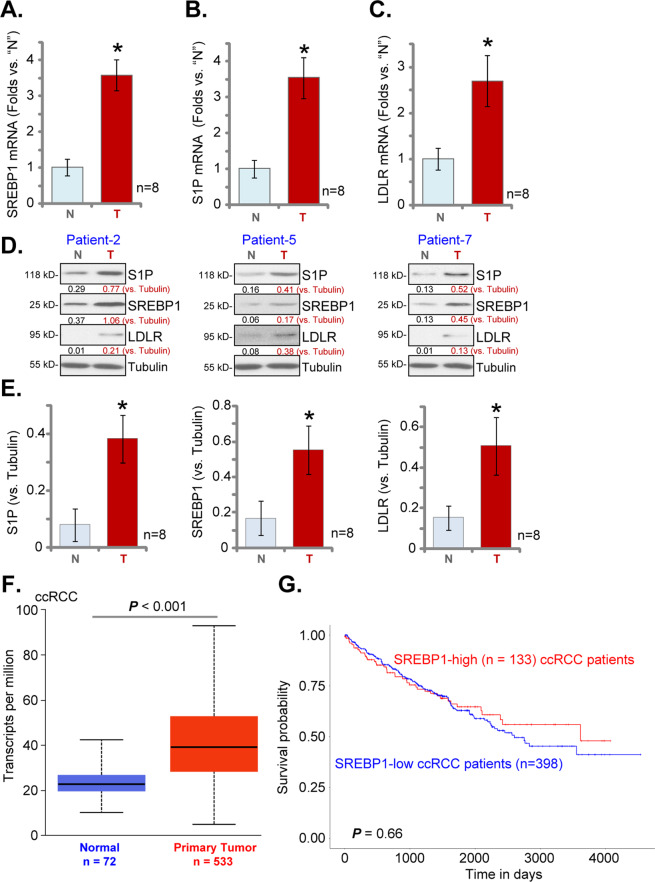
SREBP1-S1P upregulation in human RCC. Eight (*n* = 8) pairs of human RCC tumor tissues (“T”) and surrounding normal renal tissues (“N”) were obtained, expression of listed genes was tested by RT-qPCR (**A**-**C**) and Western blotting (**D**, **E**) analyses, results were normalized and quantified. The TCGA cohort shows relative *SREBP1 transcripts* in 533 cases of ccRCC tissues (“Primary Tumor”) and 72 cases of normal renal tissues (“Normal”) (**F**). Kaplan–Meier Survival analyses of *SREBP1*-low (*n* = 398, in blue) and *SREBP1*-high (*n* = 133, in red) ccRCC patients were shown (**G**). Data were expressed as the mean ± standard deviation (SD). *P < 0.05 vs. “N” tissues.

At last, we tested the expression of the SREBP1-S1P axis in human RCC. Eight (*n* = 8) pairs of human RCC tumor tissues (“T”) and surrounding normal renal tissues (“N”) were analyzed. Patients’ clinical characteristics were summarized in Table 1. As shown, *SREBP1* mRNA levels in RCC tumor tissues were significantly higher than those in the normal tissues (Fig. 6A). Furthermore, *S1P* mRNA (Fig. 6B) and *LDLR* mRNA (Fig. 6C) were upregulated in “T” tissues, while their levels were relatively low in normal tissues (Fig. 6B and C). Western blotting assays were employed to test these proteins in human tissues. Results from three representative patients, Patient-2, Patient-5, and Patient-7, demonstrated that SREBP1, S1P, and LDLR proteins were elevated in tumor tissues (Fig. 6D). Quantitative analyses integrating all eight sets of human tissues demonstrated that SREBP1, S1P, and LDLR proteins were significantly upregulated in RCC tumor tissues (*P* < 0.05 versus “N” tissues, Fig. 6E).

The Cancer Genome Atlas (TCGA) database was consulted to examine *SREBP1* expression in human ccRCC. As shown *SREBP1* transcripts in ccRCC tissues (“Primary Tumor”, *n* = 533) were significantly higher than those in the normal renal tissues (“Normal”, *n* = 72) (Fig. 6F). There was, however, no significant difference in the overall survival between *SREBP1*-high ccRCC patients and *SREBP1*-low ccRCC patients (*P* = 0.66, Fig. 6G).

## Discussion

Metabolic remodeling is a predominant feature in RCC and other cancers. It alters the utilization and/or synthesis of important metabolites, including glucose, glycogen, lipids, amino acids, and glutamine [11, 12, 39]. These rapidly proliferating cancer cells are characterized by high glucose uptake, low oxygen consumption, and elevated production of lactate, and are capable of generating fatty acids, amino acids, and nucleotides [40, 41]. These changes are important for cancer cells to retain proliferation and survival advantages under unfavorable microenvironments, as well as to invade into surrounding tissues [11, 12, 39]. Targeting key metabolic enzymes involved in the metabolic remodeling cascade may provide a novel therapeutic approach for RCC.

SREBP1 is elevated in RCC to increase lipid accumulation [11, 12]. Emerging studies have shown that, besides lipogenesis, SREBP-1-driven cancer progression could be through other mechanisms [17]. Gao et al. found that SREBP1 promoted matrix metalloproteinase 7 (MMP7) expression, NF-κB pathway activation, and colorectal cancer (CRC) cell invasion and metastasis [42]. Its expression is higher in colon adenocarcinoma [42]. Shen et al. reported SREBP1 overexpression in chemoresistant CRC. SREBP1 downregulated caspase-7 to induce gemcitabine resistance in CRC cells [43]. Zhou et al. found SREBP1 overexpression in pancreatic cancer tissues and cells [44]. High glucose-induced SREBP1 expression inhibited autophagy activation and promoted pancreatic cancer proliferation [44]. Zhang et al. showed SREBP1 overexpression in breast cancer, associated with poor prognosis. SREBP1 recruited Snail/HDAC1/2 repressor complex to inhibit E-Cadherin expression, thereby suppressing epithelial-mesenchymal transition (EMT) in breast cancer [45]. Huang et al. demonstrated that SREBP1 was essential for EMT and stemness in esophageal carcinoma [46].

Here we found that the SREBP1-S1P axis is important for RCC cell growth and proliferation. SREBP1 and S1P levels are significantly elevated in human RCC tissues. S1P silencing or CRISPR-Cas9-induced S1P KO inhibited RCC cell growth, proliferation, migration, and invasion, and provoked apoptosis activation. SREBP1 shRNA also induced RCC cell apoptosis. Conversely, ectopic overexpression of SREBP1 or S1P augmented RCC cell proliferation and migration. In vivo, silencing of SREBP1-S1P by intratumoral injection lentiviral shRNA potently inhibited RCC1 xenograft growth in SCID mice. Thus, the SREBP1-S1P axis could be an important therapeutic target for RCC.

PF-429242 is a competitive and efficient S1P inhibitor and it inhibits cholesterol synthesis [19]. PF-429242 can reversibly and competitively inhibit S1P and the expression of SREBP target genes [19]. It could also inhibit viral replication in cells infected with different viruses, including hepatitis C virus (HCV), Lassa virus, lymphocytic choriomeningitis virus, and several others [18, 47]. Here in established and primary RCC cells, PF-429242 robustly inhibited cell proliferation, and cell cycle progression, as well as cell migration and invasion. The S1P inhibitor provoked significant apoptosis activation in RCC cells. PF-429242 was however unable to induce further cytotoxicity in S1P-depleted RCC cells. Importantly, daily *i.v*. injection of a single dose of PF-429242 (10 mg/kg) potently inhibited RCC1 xenograft growth in SCID mice. These results suggest that inhibition of S1P by PF-429242 inhibited RCC cell growth in vitro and in vivo.

Several SREBP1-S1P-dependent genes are key oncogenes for RCC progression, including LDLR1, PTTG1, and ACS. Studies have implied that PTTG1 is an important oncogenic gene involved in the malignant progression of RCC [11, 12, 48, 49]. Elevated PTTG1 expression predicts poor patient prognosis in ccRCC [48, 49]. Interestingly, ectopic overexpression of PTTG1 could further promote RCC cell growth [50]. Conversely, PTTG1 silencing inhibited RCC cell proliferation and migration [49]. ACS, another SREBP1-S1P-dependent gene that is associated with tumorigenesis poor prognosis, is also upregulated in RCC [51, 52]. ACS is important for cell migration and invasion of RCC cells [51, 52]. Furthermore, Li et al. have suggested that LDLR could be a novel potential target to improve diagnosis and it can be used as an immunotherapy biomarker for RCC [8].

In this study, we found that PTTG1, LDLR, and ACS were downregulated with S1P inhibition (by PF-429242) or depletion (shRNA/KO) in RCC cells, being elevated with ectopic overexpression of S1P. Furthermore, downregulation of PTTG1, LDLR, and ACS was detected in RCC1 tumor tissues with PF-429242 injection or S1P silencing. These results further supported a key role of S1P in PTTG1, LDLR, and ACS expression.

## Conclusions

Taken together, targeting S1P by PF-429242 inhibited RCC cell growth in vitro and in vivo.

## References

[1] Siegel RL, Miller KD, Jemal A (2020). Cancer statistics, 2020. CA Cancer J Clin..

[2] Siegel RL, Miller KD, Jemal A (2019). Cancer statistics, 2019. CA Cancer J Clin..

[3] Cancer Genome Atlas Research, Network. (2013). Comprehensive molecular characterization of clear cell renal cell carcinoma. Nature..

[4] Mihaly Z, Sztupinszki Z, Surowiak P, Gyorffy B (2012). A comprehensive overview of targeted therapy in metastatic renal cell carcinoma. Curr Cancer Drug Targets..

[5] Kapoor A, Gharajeh A, Sheikh A, Pinthus J (2009). Adjuvant and neoadjuvant small-molecule targeted therapy in high-risk renal cell carcinoma. Curr Oncol..

[6] MacLennan S, Imamura M, Lapitan MC, Omar MI, Lam TB, Hilvano-Cabungcal AM (2012). Systematic review of oncological outcomes following surgical management of localised renal cancer. Eur Urol..

[7] Ljungberg B, Mehle C, Stenling R, Roos G (1996). Heterogeneity in renal cell carcinoma and its impact no prognosis-a flow cytometric study. Br J Cancer.

[8] Li F, Guo P, Dong K, Guo P, Wang H, Lv X (2019). Identification of key biomarkers and potential molecular mechanisms in renal cell carcinoma by bioinformatics analysis. J Comput Biol..

[9] Wettersten HI, Weiss RH (2013). Potential biofluid markers and treatment targets for renal cell carcinoma. Nat Rev Urol..

[10] Ljungberg B, Cowan NC, Hanbury DC, Hora M, Kuczyk MA, Merseburger AS (2010). EAU guidelines on renal cell carcinoma: the 2010 update. Eur Urol..

[11] Lee, JH, Jeon, YG, Lee, KH, Lee, HW, Park, J, Jang, H, et al. RNF20 suppresses tumorigenesis by inhibiting the SREBP1c-PTTG1 axis in kidney cancer. Mol Cell Biol. 2017;37:e00265-17. 10.1128/MCB.00265-17 PMC5660462 28827316

[12] Sethi, G, Shanmugam, MK & Kumar, AP. SREBP-1c as a molecular bridge between lipogenesis and cell cycle progression of clear cell renal carcinoma. Biosci Rep.2017;37:BSR20171270. 10.1042/BSR20171270 PMC6435460 29138263

[13] Shimano H, Sato R (2017). SREBP-regulated lipid metabolism: convergent physiology - divergent pathophysiology. Nat Rev Endocrinol..

[14] Eberle D, Hegarty B, Bossard P, Ferre P, Foufelle F (2004). SREBP transcription factors: master regulators of lipid homeostasis. Biochimie..

[15] Wang X, Sato R, Brown MS, Hua X, Goldstein JL (1994). SREBP-1, a membrane-bound transcription factor released by sterol-regulated proteolysis. Cell..

[16] Yokoyama C, Wang X, Briggs MR, Admon A, Wu J, Hua X (1993). SREBP-1, a basic-helix-loop-helix-leucine zipper protein that controls transcription of the low density lipoprotein receptor gene. Cell..

[17] Guo D, Bell EH, Mischel P, Chakravarti A (2014). Targeting SREBP-1-driven lipid metabolism to treat cancer. Curr Pharm Des..

[18] Blanchet M, Sureau C, Guevin C, Seidah NG, Labonte P (2015). SKI-1/S1P inhibitor PF-429242 impairs the onset of HCV infection. Antivir. Res.

[19] Pasquato A, Rochat C, Burri DJ, Pasqual G, de la Torre JC, Kunz S (2012). Evaluation of the anti-arenaviral activity of the subtilisin kexin isozyme-1/site-1 protease inhibitor PF-429242. Virology.

[20] Chen CM, Hsieh SC, Lin CL, Lin YS, Tsai JP, Hsieh YH (2017). Alpha-mangostin suppresses the metastasis of human renal carcinoma cells by targeting MEK/ERK expression and MMP-9 transcription activity. Cell Physiol Biochem.

[21] Wu X, Liu D, Gao X, Xie F, Tao D, Xiao X (2017). Inhibition of BRD4 Suppresses Cell Proliferation and Induces Apoptosis in Renal Cell Carcinoma. Cell Physiol Biochem..

[22] Xu M, Wang Y, Zhou LN, Xu LJ, Jin ZC, Yang DR (2020). The therapeutic value of SC66 in human renal cell carcinoma cells. Cell Death Dis..

[23] Zhu H, Mao JH, Wang Y, Gu DH, Pan XD, Shan Y (2017). Dual inhibition of BRD4 and PI3K-AKT by SF2523 suppresses human renal cell carcinoma cell growth. Oncotarget..

[24] Pan XD, Gu DH, Mao JH, Zhu H, Chen X, Zheng B (2017). Concurrent inhibition of mTORC1 and mTORC2 by WYE-687 inhibits renal cell carcinoma cell growth in vitro and in vivo. PLoS One.

[25] Wang SS, Lv Y, Xu XC, Zuo Y, Song Y, Wu GP (2019). Triptonide inhibits human nasopharyngeal carcinoma cell growth via disrupting Lnc-RNA THOR-IGF2BP1 signaling. Cancer Lett..

[26] Lv Y, Si M, Chen N, Li Y, Ma X, Yang H (2017). TBX2 over-expression promotes nasopharyngeal cancer cell proliferation and invasion. Oncotarget..

[27] Wang S, Zhong L, Li Y, Xiao D, Zhang R, Liao D (2019). Up-regulation of PCOLCE by TWIST1 promotes metastasis in Osteosarcoma. Theranostics..

[28] Zheng B, Mao JH, Qian L, Zhu H, Gu DH, Pan XD (2015). Pre-clinical evaluation of AZD-2014, a novel mTORC1/2 dual inhibitor, against renal cell carcinoma. Cancer Lett..

[29] Ye X, Ruan JW, Huang H, Huang WP, Zhang Y, Zhang F (2020). PI3K-Akt-mTOR inhibition by GNE-477 inhibits renal cell carcinoma cell growth in vitro and in vivo. Aging..

[30] Zheng J, Zhang Y, Cai S, Dong L, Hu X, Chen MB (2020). MicroRNA-4651 targets bromodomain-containing protein 4 to inhibit non-small cell lung cancer cell progression. Cancer Lett..

[31] Chen XF, Pan YS, Zheng B, Lu Q (2019). p38gamma overexpression promotes renal cell carcinoma cell growth, proliferation and migration. Biochem Biophys Res Commun..

[32] Xu XZ, Tang Y, Cheng LB, Yao J, Jiang Q, Li KR (2019). Targeting Keap1 by miR-626 protects retinal pigment epithelium cells from oxidative injury by activating Nrf2 signaling. Free Radic Biol Med..

[33] Dorotea D, Koya D, Ha H (2020). Recent insights into SREBP as a direct mediator of kidney fibrosis via lipid-independent pathways. Front Pharm..

[34] Ryan MJ, Johnson G, Kirk J, Fuerstenberg SM, Zager RA, Torok-Storb B (1994). HK-2: an immortalized proximal tubule epithelial cell line from normal adult human kidney. Kidney Int.

[35] Komoike Y, Inamura H, Matsuoka M (2012). Effects of salubrinal on cadmium-induced apoptosis in HK-2 human renal proximal tubular cells. Arch Toxicol..

[36] Wen X, Lin ZQ, Liu B, Wei YQ (2012). Caspase-mediated programmed cell death pathways as potential therapeutic targets in cancer. Cell Prolif..

[37] Chen M, Wang J (2002). Initiator caspases in apoptosis signaling pathways. Apoptosis..

[38] Porter AG, Janicke RU (1999). Emerging roles of caspase-3 in apoptosis. Cell Death Differ..

[39] Nilsson R, Jain M, Madhusudhan N, Sheppard NG, Strittmatter L, Kampf C (2014). Metabolic enzyme expression highlights a key role for MTHFD2 and the mitochondrial folate pathway in cancer. Nat Commun..

[40] Michelakis ED, Webster L, Mackey JR (2008). Dichloroacetate (DCA) as a potential metabolic-targeting therapy for cancer. Br J Cancer.

[41] Burke PJ (2017). Mitochondria, bioenergetics and apoptosis in. Cancer Trends Cancer.

[42] Gao Y, Nan X, Shi X, Mu X, Liu B, Zhu H (2019). SREBP1 promotes the invasion of colorectal cancer accompanied upregulation of MMP7 expression and NF-kappaB pathway activation. BMC Cancer.

[43] Shen W, Xu T, Chen D, Tan X (2019). Targeting SREBP1 chemosensitizes colorectal cancer cells to gemcitabine by caspase-7 upregulation. Bioengineered.

[44] Zhou C, Qian W, Li J, Ma J, Chen X, Jiang Z (2019). High glucose microenvironment accelerates tumor growth via SREBP1-autophagy axis in pancreatic cancer. J Exp Clin Cancer Res..

[45] Zhang N, Zhang H, Liu Y, Su P, Zhang J, Wang X (2019). SREBP1, targeted by miR-18a-5p, modulates epithelial-mesenchymal transition in breast cancer via forming a co-repressor complex with Snail and HDAC1/2. Cell Death Differ..

[46] Huang, CM, Huang, CS, Hsu, TN, Huang, MS, Fong, IH, Lee, WH, et al. Disruption of cancer metabolic SREBP1/miR-142-5p suppresses Epithelial-Mesenchymal transition and stemness in esophageal carcinoma. Cells. 2019;9:7. 10.3390/cells9010007 PMC7016574 31861383

[47] Uchida, L, Urata, S, Ulanday, GE, Takamatsu, Y, Yasuda, J, Morita, K, et al. Suppressive Effects of the Site 1 Protease (S1P) Inhibitor, PF-429242, on Dengue Virus Propagation. Viruses. 2016;8:46. 10.3390/v8020046 PMC4776201 26875984

[48] Wei C, Yang X, Xi J, Wu W, Yang Z, Wang W (2015). High expression of pituitary tumor-transforming gene-1 predicts poor prognosis in clear cell renal cell carcinoma. Mol Clin Oncol..

[49] Wondergem B, Zhang Z, Huang D, Ong CK, Koeman J, Hof DV (2012). Expression of the PTTG1 oncogene is associated with aggressive clear cell renal cell carcinoma. Cancer Res.

[50] Hamid T, Malik MT, Kakar SS (2005). Ectopic expression of PTTG1/securin promotes tumorigenesis in human embryonic kidney cells. Mol Cancer.

[51] Yao L, Guo X, Gui Y (2018). Acetyl-CoA synthetase 2 promotes cell migration and invasion of renal cell carcinoma by upregulating Lysosomal-associated membrane protein 1 expression. Cell Physiol Biochem.

[52] Zhang S, He J, Jia Z, Yan Z, Yang J (2018). Acetyl-CoA synthetase 2 enhances tumorigenesis and is indicative of a poor prognosis for patients with renal cell carcinoma. Urol Oncol..

